# Differentiation of the Foliar Microbiomes From Co‐Occurring Grass Species Reflects Their Recency of Invasion

**DOI:** 10.1002/ece3.74128

**Published:** 2026-08-07

**Authors:** Michael R. Fulcher

**Affiliations:** ^1^ United States Department of Agriculture, Agricultural Research Service (USDA‐ARS) Foreign Disease‐Weed Science Research Unit Frederick Maryland USA

**Keywords:** bacteria, endophytes, forest, fungi, invasive, mycobiome, North America, weeds

## Abstract

Above‐ground microbial communities associated with non‐native invasive plants are infrequently characterized, particularly during the early stages of an invasion event. This study compared the foliar fungal and bacterial microbiomes of two co‐occurring invasive grasses found in the Mid‐Atlantic United States, recently introduced *Oplismenus undulatifolius* and long‐established *Microstegium vimineum* . Amplicon sequencing was used to characterize endophytic communities collected from replicate plots within two field sites, and microbial community diversity, composition, and structure were contrasted between hosts. Despite occupying the same niche space, these invasive grasses carried significantly different microbial communities. *Oplismenus* supported lower within‐sample fungal diversity and greater among‐sample heterogeneity than *Microstegium*, while differences in bacterial communities were weaker and depended on the diversity metric examined. Null model analyses suggested that assembly processes were host‐ and microbial kingdom‐specific. The differences observed between hosts may have resulted from several non‐exclusive mechanisms but were generally consistent with an incomplete establishment of stable host‐associated symbioses by the more recent invader. Differential abundance analyses also identified multiple host‐associated fungal and bacterial lineages, including increased representation of Glomerellales, Pleosporales, and Rhizobiales in *Oplismenus*. The results of this study contribute to our understanding of co‐occurring invasive weed communities and provide insight into the formation of an emerging, invasive *Oplismenus* microbiome.

## Graphical abstract

This study examined the foliar fungal and bacterial microbiomes of two co‐occurring invasive grasses, recently introduced *Oplismenus undulatifolius* and long‐established *Microstegium vimineum* . *Oplismenus* communities exhibited lower diversity within samples and greater turnover between samples. Differences between the endophytic microbiomes of these hosts were consistent with a more recent loss of symbioses during *Oplismenus* invasion and a lag in the acquisition of new, stable associations.

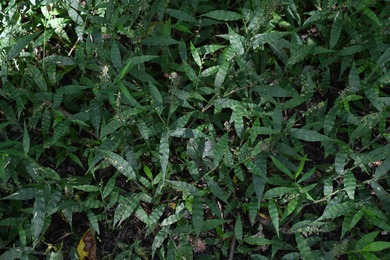

Non‐native invasive plant species interact with diverse microbes during the course of their introduction, establishment, and spread. These interactions may either facilitate or inhibit invasion success (Hardoim et al. 2015; Trognitz et al. 2016) and are therefore relevant to the management of invasive plants considered to be weeds (Cheng et al. 2022; Fulcher et al. 2024). Extensive work has been performed to understand the diversity and function of below‐ground invasive plant microbiomes (Coats and Rumpho 2014), and a growing body of literature describes above‐ground invasive plant endophytic communities (Mei et al. 2014; Fang et al. 2019; Shahrtash and Brown 2020; Lane et al. 2023; Nakamura et al. 2023; Pan et al. 2023; Bashir et al. 2024). Available evidence supports several hypotheses that explain observed patterns in the composition of invasive species microbiomes, including a loss of parasites (Keane and Crawley 2002), a loss of specialized mutualists (Moles et al. 2022), an ongoing accumulation of novel associations (Flory and Clay 2013), and the horizontal acquisition of resident native microbiomes (Fang et al. 2019), all of which connect invasion dynamics to the plant holobiont in a broad invasion‐symbiosis framework (Arnaud‐Haond et al. 2017). These patterns are often examined by comparing microbiomes in a host's native and invasive ranges (Shipunov et al. 2008; Lu‐Irving et al. 2019), or by contrasting microbiomes carried by co‐occurring invasive and native species (Aires et al. 2021; LaForgia et al. 2022).

Long‐term studies would be especially valuable for understanding how plant–microbe interactions develop and change during biological invasions, but such studies are logistically difficult and require systems with well‐documented invasion histories (Mitchell et al. 2006). In the absence of such studies, comparisons among invasive species with different residence times can be a practical, though limited, approach to observational study design. This “species‐for‐time” framework is somewhat analogous to common space‐for‐time substitutions (Pickett 1989). The substitution approach has been used to empirically test invasive plant–microbe ecology theories, and comparisons among invasive species with different introduction dates have found clear relationships between time since establishment and plant–soil feedback effects as well as pathogen accumulation (Diez et al. 2010; Mitchell et al. 2010). Although above‐ground microbiome and non‐pathogenic endophyte data remain limited, foliar fungal endophyte diversity has been shown to increase with age of populations of invasive *Ageratina adenophora* (Mei et al. 2014). Native‐ versus introduced‐range studies have also repeatedly demonstrated that plant introduction is associated with reductions in endophyte alpha diversity and altered community composition, including in *Centaurea solstitialis* (Lu‐Irving et al. 2019), *Ardisia crenata* (Nakamura et al. 2023), *Trachycarpus fortune* (Taylor et al. 1999), *Pinus* spp. (Gundale et al. 2016), *Eucalyptus* spp. (Fisher et al. 1993), and *Centaurea stoebe* (Shipunov et al. 2008). Additionally, recent work suggests that the invasive grass *Cenchrus ciliaris* associates with more generalist microbes and exhibits more variable community composition in its introduced range (Bowman et al. 2025). Together, these studies show that microbial diversity and community structure can shift during plant introduction and change over time after invasion, providing a rationale to ask whether these invasion‐associated microbiome patterns can be detected using limited species‐for‐time study designs.

To date, most studies of invasive plant microbiomes have focused on established, widely distributed invaders. Comparatively little is known about communities assembled within recently introduced plants. Examining the microbiomes of introduced plants during the early stages of their invasion may document emergent plant‐microbe associations or novel microbial diversity, allowing for characterization of incipient invasive microbiomes and potentially co‐invasive microbes (Najberek et al. 2022). Information about the identity and diversity of these microbial associates may be relevant to our ability to predict invasion success, understand microbially mediated plant competition, and maintain environmental biosecurity (Kowalski et al. 2015). Characterizing these emergent microbiomes might also lead to the discovery of novel weed biological control tools that can be rapidly developed in response to ongoing plant invasions.

In this study, foliar microbial communities formed by a relatively new invasive grass in the Mid‐Atlantic United States, *Oplismenus undulatifolius* (Ard.) Roem. & Schult. (Figure 1) (Beauchamp et al. 2013; Pham and Wu 2023), were compared to those of a frequently co‐occurring, long‐established invasive grass, *Microstegium vimineum* (Trin.) *A. Camus* (Kepner and Beauchamp 2020). *Oplismenus* was first detected in North America in 1996, whereas *Microstegium* was documented as early as 1919. This comparison assumes that time in the invaded range influences the degree to which host‐microbe associations have been acquired and stabilized. While comparing just two species, both only in the invaded range, cannot definitively separate invasion‐history effects from other host‐specific differences, this approach provides a hypothesis‐driven test for patterns expected during the course of plant invasions. *Oplismenus* was predicted to support lower within‐sample microbial diversity than *Microstegium*, consistent with a hypothesized loss of associations during introduction and a lag in the acquisition or stabilization of new symbionts. *Oplismenus* communities were further predicted to exhibit greater among‐sample turnover than *Microstegium* communities, consistent with a hypothesis that fewer conserved host–microbe associations result in a greater host sensitivity to local environmental conditions or microbial species pools.

**FIGURE 1:**
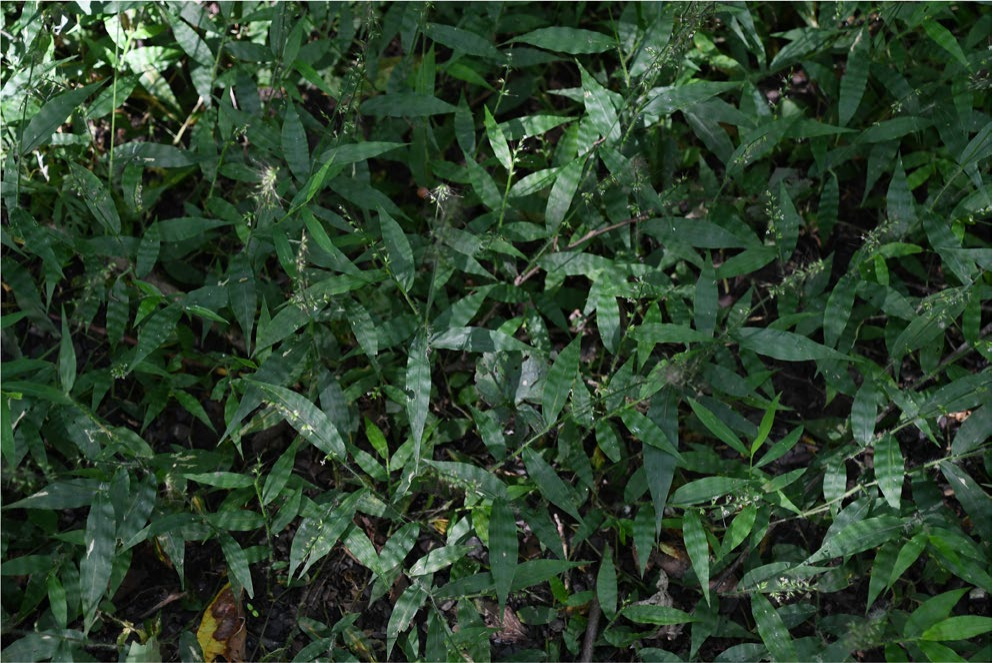
Infestation of *Oplismenus undulatifolius* in a Maryland forest understory. Wavyleaf basketgrass is a recently introduced invader of the Mid‐Atlantic United States. First detected in 1996, it continues to expand its introduced range through stolon spread and seed dispersal. Infestations form dense foliage mats in forest understory.

## 1 Materials and Methods

### 1.1 Sample Processing

Individual plant leaves were collected from two locations within the Chesapeake and Ohio National Historic Park in Maryland, USA in September 2023. The locations were separated by 90 km and were both riparian, deciduous hardwood forests. Replicate sampling plots (*n* = 4–5 per site) separated by at least 10 m were marked at each location. Sampling plots were 1 m^2^ and intentionally arranged to provide observations of naturally overlapping *Oplismenus* and *Microstegium* infestations. In all plots, each invasive grass provided approximately 50% groundcover and other plant species accounted for < 1% groundcover. The uppermost leaf from 10 randomly selected individuals of each grass species was collected using sterile tools, placed into sterile plastic containers kept on ice, and transported for laboratory processing. Leaves were rinsed in sterile distilled H_2_O, surface disinfested by agitating in 0.825% NaOCl for 15 min, and triple rinsed in sterile distilled H_2_O. Successful disinfestation of leaf surfaces was confirmed by plating the final rinsate on Petri plates containing potato dextrose agar (PDA) and observing incubated plates for microbial growth. Disinfested leaves were air dried to remove residual rinsate and frozen at −80°C.

### 1.2 Sequence Analysis

Foliar endophyte community DNA was purified from leaves using the Zymo Quick‐DNA Fungal/Bacterial Kit (Zymo Research, Irvine). A randomly selected subset of seven leaf extracts from each plot and species was ultimately selected for sequencing (*n* = 63 leaves per species). Three blank DNA extracts using sterile water as an input were included as negative controls to monitor for laboratory contaminants (Hornung et al. 2019). Library preparation and Illumina sequencing were performed at the University of Minnesota Genomics Center. Amplicons of the fungal internal transcribed spacer region of ribosomal DNA (ITS 2 primers: 5.8SR TCGATGAAGAACGCAGCG and ITS4 TCCTCCGCTTATTGATATGC) and of the bacterial 16S ribosomal DNA (16S V4 primers: 515F GTGCCAGCMGCCGCGGTAA and 806R GGACTACHVGGGTWTCTAAT) were sequenced on an Illumina NextSeq 2000 outputting 2 × 300 bp reads (White et al. 1990; Caporaso et al. 2011; Gohl et al. 2016). Sequences were trimmed and denoised using ‘cutadapt’ and ‘dada2’ as implemented in QIIME2 v2024.2 following visual examination of read quality plots (Martin 2011; Callahan et al. 2016; Bolyen et al. 2019). The resulting exact sequence variants were clustered into operational taxonomic units (OTUs) at a 99% identity level with ‘vsearch’ (Rognes et al. 2016). Taxonomy was assigned to 16S and ITS OTUs using naïve Bayesian classifiers trained on the Greengenes2 “gg_2022_10_backbone_full_length” database and the UNITE V10 “99_all_04” database, respectively (Pedregosa et al. 2011; Abarenkov et al. 2023; McDonald et al. 2024). OTUs were filtered by taxonomic assignment to remove 16S sequences originating from mitochondria, chloroplasts, eukaryotes, and archaea as well as ITS sequences originating from protists and plants.

OTU abundances and taxonomic assignments were exported for statistical analysis in R using the RStudio environment (R Core Team 2025; Posit Team 2025). Alpha diversity was calculated using Hill numbers of order 0, 1, and 2—corresponding to richness, exponential Shannon's diversity, and inverse Simpson's diversity—and compared between grass species using rarefied point estimates generated with the ‘iNext’ package (Hsieh et al. 2016; Schloss 2024). For both 16S and ITS data, average point estimates of diversity were calculated from 100 replicate subsamples at depths ranging from 1 to 1900 sequencing reads. For each rarefaction depth and Hill order, diversity estimates were analyzed separately using linear mixed‐effects models implemented in the ‘lme4’ package with log‐transformed effective OTU number as the response, host species as a fixed effect, and plot nested within location as a random effect (Bates et al. 2015). This structure accounted for non‐independence among leaves collected from the same plots and locations. Host‐level estimated marginal means were calculated from each model using the ‘emmeans’ package and back‐transformed to the original diversity scale (Lenth and Piaskowski 2026). Pairwise host contrasts were then obtained with the *pairs* function for each depth × Hill‐order combination. Following this, all subsequent analyses used a rarefaction depth of 400 reads, since this threshold allowed for the retention of 122 ITS and 120 16S samples. Beta‐diversity was calculated as Bray‐Curtis (B‐C) dissimilarity between samples, and the *avgdist* function from the ‘vegan’ package was used to generate mean B‐C dissimilarities based on 1000 replicate samplings at a depth of 400 sequences (Oksanen et al. 2026). Distance based redundancy analysis (dbRDA) was performed with location, plot, and plant species as nested predictors to visualize and test for differentiation in beta‐diversity between grass microbiomes. The significance of each predictor was calculated with a permutational analysis of variance run with 999 permutations. Sample distances to group medians were contrasted with non‐parametric Wilcoxon rank‐sum tests, and spatial autocorrelation was assessed with Mantel tests comparing sample physical distance to microbiome B‐C dissimilarity. To evaluate whether community turnover was more consistent with stochastic or deterministic assembly processes, modified taxonomic stochasticity ratios were calculated using the ‘NST’ package with the null model assumptions that colonization is proportional to relative abundance and that total richness is held constant in each community (Ning et al. 2019; Liang et al. 2020). Stochasticity in relative abundance structure was differentiated from stochasticity in taxon membership by running the analysis with both an abundance weighted distance measure (Bray‐Curtis) and a corresponding unweighted distance measure (Sorenson). Bootstrap testing was performed using the *nst.boot* function to compare host species with 1000 resamples. Lastly, differences in microbiome composition were detected using Analysis of Compositions of Microbiomes with Bias Correction (ANCOM‐BC, implemented in QIIME2) to examine the relative abundance of individual OTUs (Lin and Peddada 2020).

## 2 Results

After processing, ITS sequence counts per sample ranged from 101 to 545,106 (*Microstegium* median = 1978; *Oplismenus* median = 209,328). A total of 389 fungal OTUs were recorded, with individual samples containing between 6 and 70 OTUs (*Microstegium* median = 19; *Oplismenus* median = 22). 16S sequence counts ranged from 274 to 124,602 per sample (*Microstegium* median = 700; *Oplismenus* median = 14,315). A total of 1831 bacterial OTUs were recorded, with individual samples containing between 12 and 221 OTUs (*Microstegium* median = 25; *Oplismenus* median = 59). The substantial variation in sequencing depth may be attributed to the physical structure of host leaves, the total abundance of microbes in each endophytic community, or host‐specific differences in the efficiency of DNA extractions and library preparations. A rarefaction‐based approach to analysis was used to normalize sequencing depths.

**FIGURE 2:**
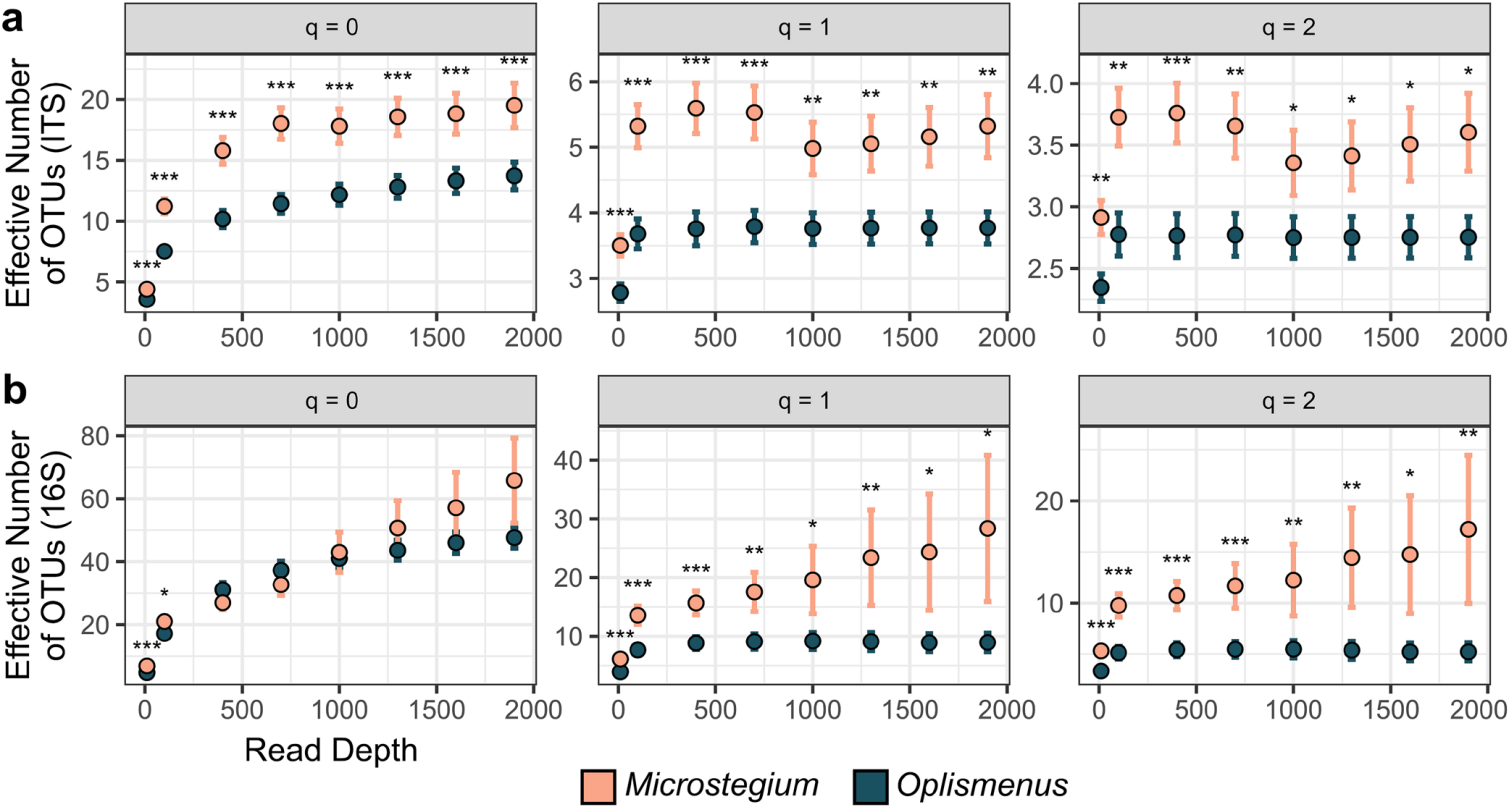
Sequence diversity of foliar endophytic communities from *Oplismenus* and *Microstegium*. Estimated marginal means for (a) fungal ITS and (b) bacterial 16S communities are shown across multiple rarefaction depths. Points represent estimated marginal means from linear mixed‐effects models with log‐transformed diversity as the response, host as a fixed effect, and plot nested within location as a random effect. Error bars indicate standard errors. Asterisks denote significant pairwise contrasts between hosts at a given rarefaction depth (*p < 0.05; **p < 0.01; ***p < 0.001). Panels show results for Hill numbers *q* = 0 (richness), *q* = 1 (exponential Shannon Diversity), and *q* = 2 (inverse Simpson Diversity). Shading denotes host species.

The alpha diversity of fungal and bacterial communities differed between *Oplismenus* and *Microstegium*. Rarefaction curves indicated that *Microstegium* generally supported higher ITS and 16S OTU diversity than *Oplismenus* across the sequencing depths examined (Appendix Figure S1). To test these patterns while accounting for the nested sampling design, rarefied diversity estimates were modeled separately across read depths and Hill number orders using linear mixed models. These models showed that host identity significantly affected fungal alpha diversity at all rarefaction depths and for all Hill number orders tested (*χ* ^2^ ≥ 4.28, *p* ≤ 0.039) (Appendix Tables S1 and S2). Bacterial alpha diversity showed a more conditional response. Host effects on richness, *q* = 0, were not significant at rarefaction depths of 400 reads or greater, whereas host effects for *q* = 1 and *q* = 2 remained significant across all depths (*χ* ^2^ ≥ 6.01, *p* ≤ 0.014). Estimated marginal means from these models confirmed that *Microstegium* had higher fungal diversity than *Oplismenus* across all Hill orders and depths, while bacterial differences were strongest for diversity metrics that give decreasing weight to rare taxa (Figure 2). This indicates that bacterial differences between hosts were driven more by differences in evenness or dominance structure than by richness alone.

**FIGURE 3:**
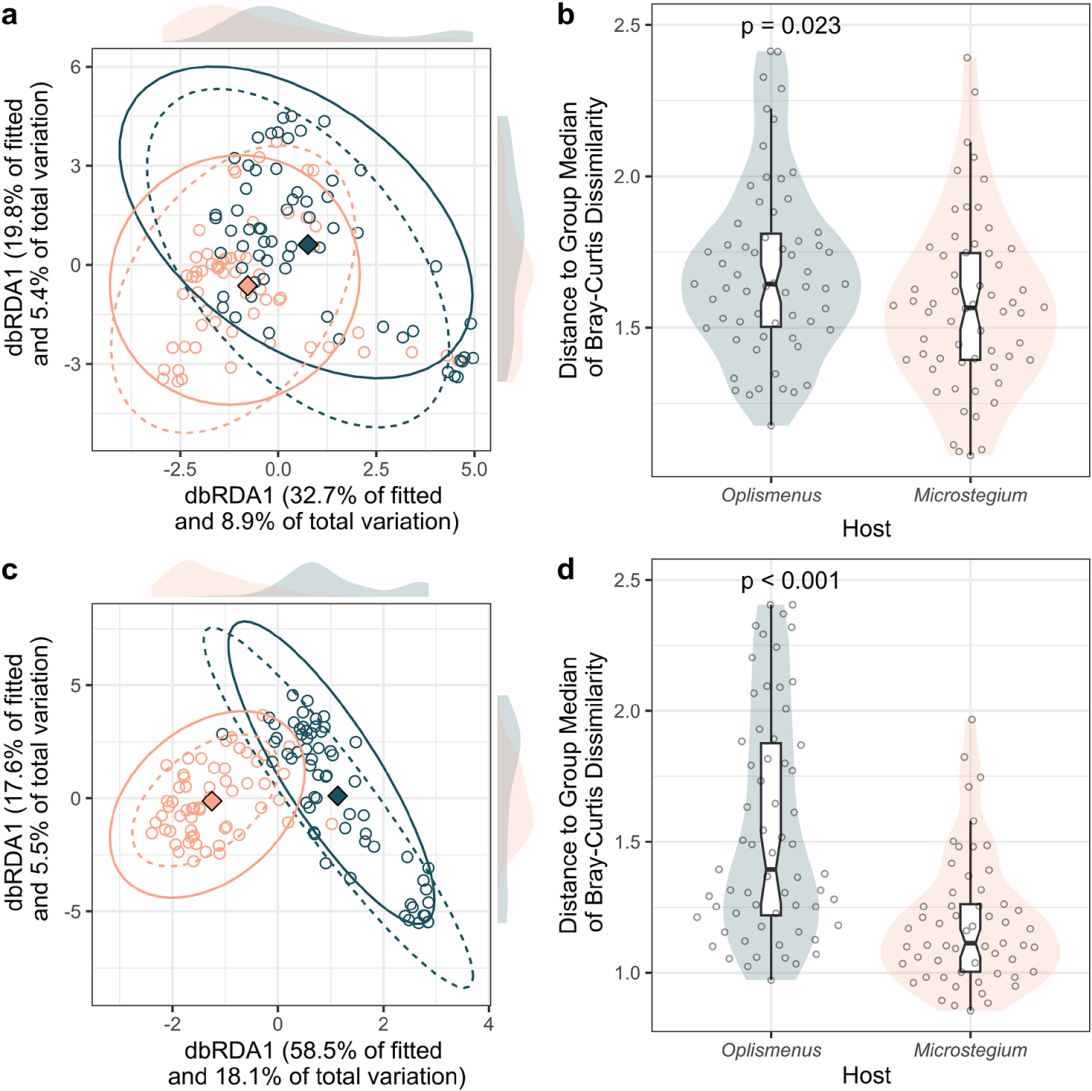
Dissimilarity of *Oplismenus* and *Microstegium* foliar endophytic communities. The average Bray–Curtis dissimilarity of sequence relative abundances repeatedly rarefied to a depth of 400 reads for (a) fungal and (c) bacterial communities was visualized through a distance‐based redundancy analysis. Each circular point represents a single leaf, and host medians are indicated by a filled diamond. Ellipses mark 95% confidence intervals around each host, and line type corresponds to the field site where samples were collected. The dispersion of (b) fungal and (d) bacterial communities around host medians was significantly different according to Wilcoxon rank‐sum tests and is displayed as an interquartile range with median marked by a horizontal line. Shading denotes host species.

In dbRDA and permutational analyses, host identity explained the largest assigned portion of variation in both fungal and bacterial communities (Figure 3a,c). Host‐associated differentiation was stronger than location‐level differentiation for both marker datasets, and plot‐level effects also contributed significantly to community structure (Table 1). Community dispersion differed between hosts, with *Oplismenus* communities showing greater dispersion around host group medians than *Microstegium* communities for both fungal and bacterial datasets (Figure 3b,d). This indicates that foliar microbiomes from *Oplismenus* were more heterogeneous among individual leaves, whereas *Microstegium* communities were more compositionally consistent. Mantel tests further suggested that fungal community dissimilarity was spatially structured in both hosts, but spatial autocorrelation was stronger in *Oplismenus* than in *Microstegium* (Table 2). Modified stochasticity ratios indicated that host‐associated assembly patterns differed between fungal and bacterial communities and depended on whether abundance structure or taxon membership was emphasized (Table 3). For fungal communities, abundance‐weighted MST was significantly higher in *Oplismenus* than in *Microstegium*. This suggests that the relative abundance structure of fungal communities in *Oplismenus* was more stochastic. In contrast, unweighted fungal MST did not differ significantly between hosts, indicating no clear evidence that fungal taxon membership was more stochastic in either host. Based on these results, *Oplismenus* does not necessarily recruit a more random set of fungal taxa, but the taxa that become abundant vary more stochastically among samples. For bacterial communities, MST was significantly higher in *Microstegium* in both abundance‐weighted and unweighted analyses. It appears that *Microstegium* bacterial communities were therefore more stochastic in both relative abundance structure and taxon membership. Together, these beta‐diversity analyses show that host identity was a major driver of foliar microbiome composition, that *Oplismenus* microbiomes were more heterogeneous among samples, and that this heterogeneity could reflect multiple forces, including spatial structuring, host filtering, and differences between fungal and bacterial assembly processes.

**TABLE 1:** Permutational tests partitioned *Oplismenus* and *Microstegium* foliar microbiome dissimilarity among potential sources of variation.

	df^a^	SS^b^	*F*	*p*	*R* ^2^
Fungi					
Location	1	10.961	4.443	0.001	0.031
Plot	7	28.820	1.669	0.004	0.082
Host	9	55.788	2.513	0.001	0.158
Residuals	104	256.554			
*Bacteria*					
Location	1	2.321	1.174	0.25	0.008
Plot	7	24.540	1.773	0.005	0.084
Host	9	63.412	3.563	0.001	0.217
Residuals	102	201.701			

^a^ Degrees of freedom.

^b^ Sum of squares.

**TABLE 2:** Mantel tests differentiated the levels of spatial autocorrelation found in foliar microbial communities inhabiting *Oplismenus* and *Microstegium*.

	Mantel *r*	*p*
*Oplismenus*		
Fungi	0.157	0.002
Bacteria	0.038	0.062
*Microstegium*		
Fungi	0.074	0.008
Bacteria	−0.004	0.486

**TABLE 3:** Modified stochastic ratios for foliar endophytic communities in *Oplismenus* and *Microstegium*.

Marker	Distance metric^a^	*Microstegium* MST^b^	*Oplismenus* MST	*p* ^c^
ITS	Bray‐Curtis	0.256	0.339	0.008
ITS	Sørenson	0.563	0.525	0.282
16S	Bray‐Curtis	0.598	0.412	< 0.001
16S	Sørenson	0.600	0.515	0.009

^a^ Bray–Curtis dissimilarity incorporates relative abundance data so that MST provides information about abundance structure, and Sørenson dissimilarity is based on taxa presence and absence, providing information about occurrence structure.

^b^ MST = modified stochasticity ratio, ranging from 1 (stochastic) to 0 (deterministic).

^c^ Bootstrap comparisons calculated from 1000 replicates.

**FIGURE 4:**
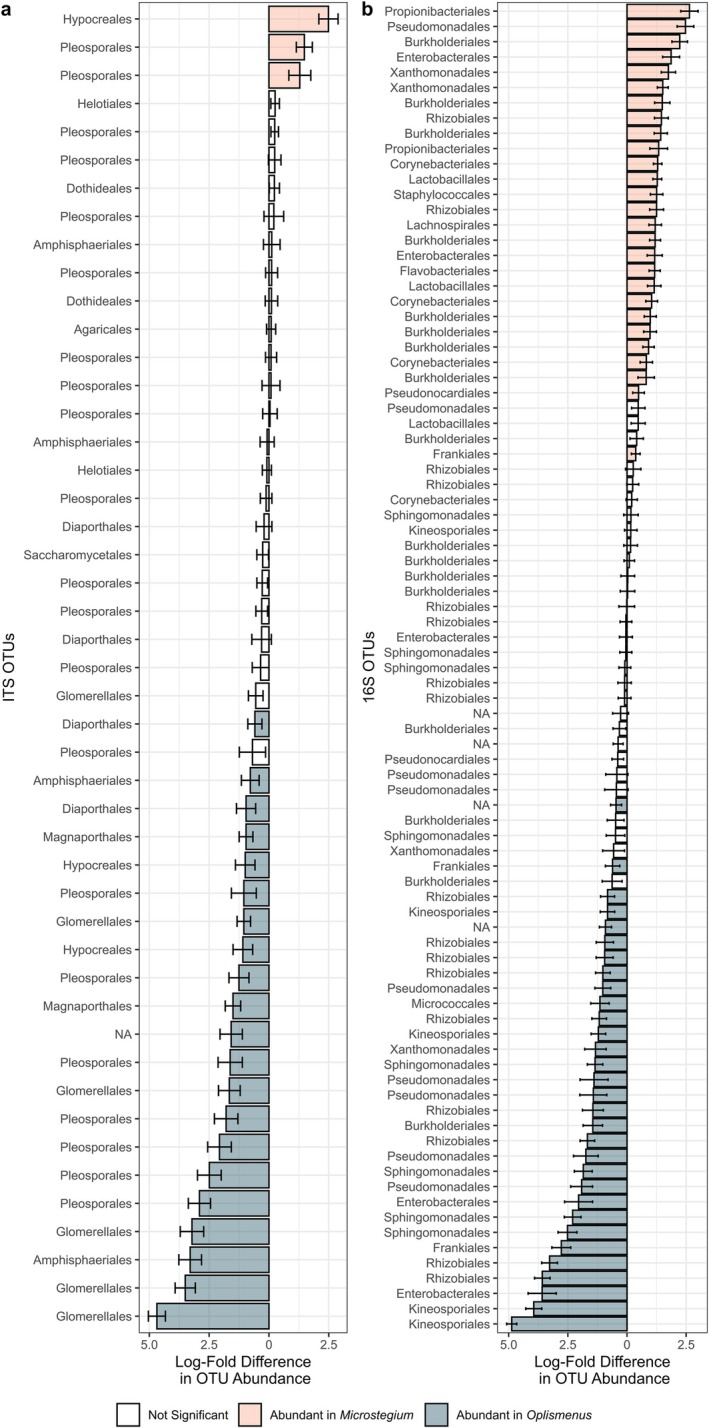
Differential relative abundance of sequences shared between *Oplismenus* and *Microstegium*. Log‐fold changes in the relative abundance of individual (a) fungal ITS2 and (b) bacterial 16S OTUs from each host are plotted by magnitude of change and labeled by taxonomic Order. Bars indicate the mean log‐fold difference between hosts, and error bars mark 95% confidence intervals. Shading denotes host species and significance at *p* < 0.05 calculated using Analysis of Compositions of Microbiomes with Bias Correction.

Individual ITS and 16S OTUs were detected that differed in relative abundance between the two hosts according to ANCOM‐BC (Figure 4). Differentially abundant fungal OTUs were distributed across several taxonomic orders, and OTUs assigned to Glomerellales and Pleosporales were among those most frequently found more abundant in *Oplismenus*. Differentially abundant bacterial OTUs likewise spanned multiple lineages, including a marked increase in the relative abundance of Rhizobiales in *Oplismenus*. These host‐associated differences suggest that the two grasses support distinct microbial assemblages not only at the level of whole‐community diversity and composition, but also in representation of individual fungal and bacterial taxa.

## 3 Discussion

Co‐occurring populations of invasive *Oplismenus* and *Microstegium* possessed substantially different foliar endophytic microbiomes, despite both being warm season grasses that invade the same environments. By reporting on substantial differences in the diversity, composition, and structure of foliar microbiomes inhabiting these two grasses, this study improves our understanding of the biology of co‐occurring invasive species (Kuebbing et al. 2013). *Oplismenus* carried microbiomes containing less within‐sample diversity than *Microstegium* microbiomes. This finding is consistent with *Oplismenus* losing symbiotic associations during a more recent introduction event and experiencing a lag in new symbiont acquisition, supporting expectations for the enemy removal and lost mutualist hypotheses (Enders et al. 2020). This would also be congruent with observations about natural enemy associations in the co‐invaded range. In contrast to *Oplismenus*, which has no disease occurrence records in North America (Farr and Rossman 2019), *Microstegium* has been established in the invaded range long enough to accumulate multiple plant pathogens (Stricker et al. 2016).

While losses of host–microbe associations and decreases in endophyte richness have been repeatedly documented for introduced or invasive plants, patterns of microbial turnover among aboveground endophytes from invasive plants remain less clearly resolved. In this study, the more recent invader, *Oplismenus*, exhibited greater dispersion of community dissimilarities than the longer‐established *Microstegium*. Several non‐exclusive mechanisms could explain this pattern. For instance, recent invaders may be unable to impose strong host filters on microbes in the invaded range or may act as promiscuous hosts (Bonthond et al. 2021). Generally, these explanations might result in signatures of a community assembly process driven by more stochastic rather than deterministic processes, similar to those occurring in systems experiencing disturbance that results in de‐regulation of host‐associated microbes (Arnault et al. 2023). Modified stochasticity ratio analyses provided only partial support for these interpretations but also showed that the stochastic signal was dependent on microbial kingdom, emphasizing the likelihood of multiple interacting forces driving heterogeneity in *Oplismenus* endophyte communities.

Greater turnover in *Oplismenus* could also reflect heightened sensitivity to local environmental conditions or microbial species pools in the absence of stable communities of co‐evolved symbionts. The spatial autocorrelation observed suggests that *Oplismenus* microbiome differentiation may be related to local environmental variation or spatially structured inoculum availability. If *Oplismenus* is more responsive to these local influences, individual leaves or ramets may acquire different subsets of available microbes, producing high beta‐dispersion. The perennial, stoloniferous growth of *Oplismenus* may also expose connected plants or ramets to a greater variety of microhabitats over time, increasing the number of local niches from which microbes could be acquired. Another explanation could be that *Oplismenus* communities are more strongly shaped by priority effects, where early colonists become dominant and competitively exclude other fungi. If the identity of those dominant residents varies among microenvironments, this might produce both low alpha diversity and high dispersion. This would also be consistent with the MST results showing fungal abundance structure in particular could be explained by stochastic processes. While some of the above mechanisms might be related to host‐specific differences between *Oplismenus* and *Microstegium*, including phenology and morphology, they are also consistent with expectations for a newly introduced invader with few conserved microbial associates. As the *Oplismenus* invasion progresses, future work may detect more stable and conserved host–microbe associations that result in less microbiome heterogeneity and suppress the influence of local species pools, priority effects, and other context‐dependent assembly processes. Interestingly, the foliar patterns from the present study also contrast to some extent with below‐ground microbial interactions that indicated *Oplismenus* may be amplifying specific environmental taxa, increasing local diversity, and homogenizing resident soil microbiomes (Fulcher et al. 2026). This contrast suggests that Oplismenus structures above‐ and below‐ground microbial communities through different mechanisms, and that inferences from below‐ground invasive plant‐microbe ecology might not transfer clearly to the above‐ground host environment.

Horizontal acquisition of microbial associates by a new invader may be possible during range expansion as newly introduced plants encounter phylogenetically related host species carrying compatible microbiota. In particular, the movement of fungal pathogens between resident and introduced plants would seem to be a likely occurrence (Chen et al. 2020). Because *Oplismenus* and *Microstegium* plants sampled in this study were found growing in adjacent infestations, resident microbes from established *Microstegium* populations were expected to be shared with *Oplismenus*. However, of the 389 fungal OTUs recorded, only 47 were found in both hosts. This is evidence that the two hosts share only a small fraction of their fungal microbiomes and contrasts with findings from a study of native and long‐established exotic grasses in New Zealand that found extensive sharing of foliar fungal endophytes (Visscher and Dickie 2021).

Foliar endophytic communities in *Oplismenus* contained a marked increase in the relative abundance of OTUs classified to the fungal orders Glomerellales and Pleosporales and the bacterial order Rhizobiales, as compared to *Microstegium* communities. Future work characterizing individual microbes from *Oplismenus* might focus on taxa in these lineages as they could represent important or host‐specific components of the emerging invasive *Oplismenus* microbiome. The high relative abundance of Glomerellales in *Oplismenus* is notable since a previous amplicon sequencing‐based study of *Microstegium* foliar fungal communities did not record any sequences assigned to this order (Lane et al. 2023). Pleosporalean fungi encompass a large, diverse group of species, and the differential relative abundances of specific sequence variants observed within the lineage suggest *Oplismenus* and *Microstegium* are each preferentially colonized by related but distinct sets of taxa. Members of the Rhizobiales are best known as root symbionts that form nitrogen‐fixing nodules in leguminous plants. However, these bacteria have been reported previously as a component of foliar rice microbiomes, where they were found to enhance growth traits including root and shoot biomass, photosynthetic rates, and water use efficiency (Chi et al. 2005). The high relative abundance of Rhizobiales recorded in *Oplismenus* leaves raises the possibility that these associations are promoting the competitive ability of the newer invader, possibly contributing to its ability to colonize locations that have previously contained only dense monocultures of *Microstegium*. This could help explain the co‐existence of both species in the same forest understory niche despite *Oplismenus* being a comparatively weaker competitor under experimentally shaded greenhouse conditions (Kepner and Beauchamp 2020). The ability of foliar plant pathogens to influence the interspecific competitive ability of invasive plants has been shown repeatedly (Kendig et al. 2021; Fulcher and Little 2024), and while phyllosphere microbes can be considered primarily consumers whose removal leads to increased plant productivity (Lane et al. 2025), there are likely complex plant–microbe–microbe interactions governing the effects of phyllosphere symbioses on plant productivity (Almeida et al. 2024). There is a continuing need to determine how such interactions between above‐ground microbial associates might influence the success and control of plant invasions.

Plant invasions can be managed through the use of biological control agents, sometimes including plant pathogenic microbes. Existing approaches to microbial‐based invasive plant control require the identification and development of either (i) host‐specific native range plant pathogens for safe importation and release or (ii) highly damaging plant pathogens already present in the invaded range that are suitable for development into inundatively applied bioherbicides (Morin 2020). The utility of biological control agents is severely constrained by their years‐to‐decades long development timelines, which exclude them from rapid deployment during the initial stages of plant invasions when interventions have a high impact on invasion trajectory and outcomes (Hoddle 2024; Smith et al. 2025). Research pipelines that develop biological control tools for use during rapid responses to invasion are therefore desirable, and timely characterization of microbes associated with invasive plant species would be essential to permit such an approach. The present study may provide foundational information required to guide the collection and screening of microbes for plant suppressive activity as the *Oplismenus* invaded range continues to expand.

## 4 Conclusions

The foliar endophytic microbiome of a recently introduced invasive grass, *Oplismenus undulatifolius*, was contrasted with that of a long‐established co‐occurring invader, *Microstegium vimineum*, to test whether patterns predicted by microbial ecology and invasion theory were detectable through a limited species‐for‐time comparison. Consistent with predictions, *Oplismenus* exhibited lower within‐sample fungal diversity and greater among‐sample turnover than *Microstegium*, though differences between bacterial communities were less strongly evidenced. Host identity was the major determinant of community composition for both fungi and bacteria, and results suggest that the foliar microbiome of *Oplismenus* is comparatively less conserved and more dependent on local contexts, possibly reflecting a combination of incomplete establishment of host‐associated symbioses, sensitivity to variation in species pools or environmental conditions, and stochastically driven community abundance structure. Although this invaded‐range comparison of two grasses cannot separate invasion‐history effects from other host‐specific differences, it establishes a baseline of foliar endophytic diversity associated with an emerging invader and identifies putative taxa and lineages (e.g., Glomerellales, Pleosporales, and Rhizobiales) that differ in relative abundance between hosts. Together, these findings should motivate future work that links foliar community patterns to functional outcomes, tests whether *Oplismenus* microbiomes become more conserved as residence time increases, and evaluates whether specific microbial associates contribute to invasion dynamics or offer targets for microbial‐based management.

## Author Contributions

**Michael R. Fulcher:** conceptualization (equal), data curation (equal), formal analysis (equal), investigation (equal), methodology (equal), project administration (equal), visualization (equal), writing – original draft (equal).

## Funding

This work was supported by Agricultural Research Service, 0201‐88888‐002‐000D, 0201‐88888‐003‐000D, 8044‐30400‐001‐000‐D.

## Conflicts of Interest

The author declares no conflicts of interest.

## Supporting information


**Figure S1:** Rarefaction curves for foliar microbial diversity in *Oplismenus* and *Microstegium*. Diversity of (a) fungal (ITS) and (b) bacterial (16S) sequences from endophytic communities in basketgrass and stiltgrass leaves. The effective number of OTUs, or Hill numbers of order *q* = 0 (richness), *q* = 1 (exponential Shannon Diversity), and *q* = 2 (inverse Simpson Diversity), were estimated across a range of rarefaction depths based on bootstrap resampling (*n* = 100 per depth). Each line represents the community from a single leaf. Shading denotes host.

**Table S1:** Summary of analysis of variance to test for the significance of host effect in mixed linear models of microbial alpha diversity.
**Table S2:** Summary of linear mixed model parameter estimates for alpha diversity across rarefaction depths.

## Data Availability

Sequencing data are available from NCBI GenBank. Demultiplexed fastq files are deposited under BioProject PRJNA1245354, Samples SAMN47750341‐SAMN47750466. All additional data, metadata, and analysis scripts are publicly available through USDA AgDataCommons: https://doi.org/10.15482/USDA.ADC/31061641.

## References

[ece374128-bib-0001] Abarenkov, K. , R. H. Nilsson , K.‐H. Larsson , et al. 2023. “The UNITE Database for Molecular Identification and Taxonomic Communication of Fungi and Other Eukaryotes: Sequences, Taxa and Classifications Reconsidered.” Nucleic Acids Research 52: D791–D797. 10.1093/nar/gkad1039. PMC10767974 37953409

[ece374128-bib-0002] Aires, T. , T. M. Stuij , G. Muyzer , E. A. Serrão , and A. H. Engelen . 2021. “Characterization and Comparison of Bacterial Communities of an Invasive and Two Native Caribbean Seagrass Species Sheds Light on the Possible Influence of the Microbiome on Invasive Mechanisms.” Frontiers in Microbiology 12: 653998. 10.3389/fmicb.2021.653998. 34434172 PMC8381869

[ece374128-bib-0003] Almeida, B. K. , E. H. Tran , and M. E. Afkhami . 2024. “Phyllosphere Fungal Diversity Generates Pervasive Nonadditive Effects on Plant Performance.” New Phytologist 243: 2416–2429. 10.1111/nph.19792. 38719779

[ece374128-bib-0004] Arnaud‐Haond, S. , T. Aires , R. Candeias , et al. 2017. “Entangled Fates of Holobiont Genomes During Invasion: Nested Bacterial and Host Diversities in *Caulerpa taxifolia* .” Molecular Ecology 26: 2379–2391. 10.1111/mec.14030. 28133884

[ece374128-bib-0005] Arnault, G. , C. Mony , and P. Vandenkoornhuyse . 2023. “Plant Microbiota Dysbiosis and the Anna Karenina Principle.” Trends in Plant Science 28: 18–30. 10.1016/j.tplants.2022.08.012. 36127241

[ece374128-bib-0072] Bashir, I. , A. F. War , I. Rafiq , Z. A. Reshi , I. Rashid , and Y. S. Shouche . 2024. “Uncovering the Secret Weapons of an Invasive Plant: The Endophytic Microbes of *Anthemis cotula* .” Heliyon 10, no. 9: e29778. 10.1016/j.heliyon.2024.e29778. 38694109 PMC11058297

[ece374128-bib-0006] Bates, D. , M. Mächler , B. Bolker , and S. Walker . 2015. “Fitting Linear Mixed‐Effects Models Using lme4.” Journal of Statistical Software 67: 1–48. 10.18637/jss.v067.i01.

[ece374128-bib-0007] Beauchamp, V. B. , S. M. Koontz , C. Suss , et al. 2013. “An Introduction to *Oplismenus undulatifolius* (Ard.) Roem. & Schult. (Wavyleaf Basketgrass), a Recent Invader in Mid‐Atlantic Forest Understories.” Journal of the Torrey Botanical Society 140: 391–413. 10.3159/TORREY-D-13-00033.1.

[ece374128-bib-0008] Bolyen, E. , J. R. Rideout , M. R. Dillon , et al. 2019. “Reproducible, Interactive, Scalable and Extensible Microbiome Data Science Using QIIME 2.” Nature Biotechnology 37: 852–857. 10.1038/s41587-019-0209-9. PMC7015180 31341288

[ece374128-bib-0009] Bonthond, G. , T. Bayer , S. A. Krueger‐Hadfield , et al. 2021. “The Role of Host Promiscuity in the Invasion Process of a Seaweed Holobiont.” ISME Journal 15: 1668–1679. 10.1038/s41396-020-00878-7. 33479490 PMC8163768

[ece374128-bib-0010] Bowman, E. A. , C. V. Hawkes , N. Jones , R. M. Plowes , D. J. Martins , and L. E. Gilbert . 2025. “Invasive Buffelgrass, *Cenchrus ciliaris* , Balances Opportunistic Acquisition of Foliar Fungi With Host and Environmental Filtering in Its Introduced Range.” Molecular Ecology 34: e17609. 10.1111/mec.17609. 39665802 PMC11701881

[ece374128-bib-0011] Callahan, B. J. , P. J. McMurdie , M. J. Rosen , et al. 2016. “DADA2: High‐Resolution Sample Inference From Illumina Amplicon Data.” Nature Methods 13: 581–583. 10.1038/nmeth.3869. 27214047 PMC4927377

[ece374128-bib-0012] Caporaso, J. G. , C. L. Lauber , W. A. Walters , et al. 2011. “Global Patterns of 16S rRNA Diversity at a Depth of Millions of Sequences Per Sample.” Proceedings of the National Academy of Sciences 108: 4516–4522. 10.1073/pnas.1000080107. PMC3063599 20534432

[ece374128-bib-0013] Chen, L. , J. Zhou , T. Zeng , et al. 2020. “Quantifying the Sharing of Foliar Fungal Pathogens by the Invasive Plant *Ageratina adenophora* and Its Neighbours.” New Phytologist 227: 1493–1504. 10.1111/nph.16624. 32343409

[ece374128-bib-0014] Cheng, L. , A. Ditommaso , J. Kao‐kniffin , and J. Kao‐kniffin . 2022. “Opportunities for Nicrobiome Suppression of Weeds Using Regenerative Agricultural Technologies.” 2: 1–13. 10.3389/fsoil.2022.838595.

[ece374128-bib-0015] Chi, F. , S.‐H. Shen , H.‐P. Cheng , Y. X. Jing , Y. G. Yanni , and F. B. Dazzo . 2005. “Ascending Migration of Endophytic Rhizobia, From Roots to Leaves, Inside Rice Plants and Assessment of Benefits to Rice Growth Physiology.” Applied and Environmental Microbiology 71: 7271–7278. 10.1128/AEM.71.11.7271-7278.2005. 16269768 PMC1287620

[ece374128-bib-0016] Coats, V. C. , and M. E. Rumpho . 2014. “The Rhizosphere Microbiota of Plant Invaders: An Overview of Recent Advances in the Microbiomics of Invasive Plants.” Frontiers in Microbiology 5: 368. 10.3389/fmicb.2014.00368. 25101069 PMC4107844

[ece374128-bib-0017] Diez, J. M. , I. Dickie , G. Edwards , P. E. Hulme , J. J. Sullivan , and R. P. Duncan . 2010. “Negative Soil Feedbacks Accumulate Over Time for Non‐Native Plant Species.” Ecology Letters 13: 803–809. 10.1111/j.1461-0248.2010.01474.x. 20482584

[ece374128-bib-0018] Enders, M. , F. Havemann , F. Ruland , et al. 2020. “A Conceptual Map of Invasion Biology: Integrating Hypotheses Into a Consensus Network.” Global Ecology and Biogeography 29: 978–991. 10.1111/geb.13082. 34938151 PMC8647925

[ece374128-bib-0070] Fang, K. , Y.‐F. Miao , L. Chen , et al. 2019. “Tissue‐Specific and Geographical Variation in Endophytic Fungi of *Ageratina adenophora* and Fungal Associations With the Environment.” Frontiers in Microbiology 10. 10.3389/fmicb.2019.02919. PMC6930192 31921082

[ece374128-bib-0020] Farr, D. F. , and A. Y. Rossman . 2019. Fungal Databases, U.S. National Fungus Collections, ARS, USDA.

[ece374128-bib-0021] Fisher, P. J. , O. Petrini , and B. C. Sutton . 1993. “A Comparative Study of Fungal Endophytes in Leaves, Xylem and Bark of Eucalyptus in Australia and England.” Sydowia 45: 338–345.

[ece374128-bib-0022] Flory, S. L. , and K. Clay . 2013. “Pathogen Accumulation and Long‐Term Dynamics of Plant Invasions.” Journal of Ecology 101: 607–613. 10.1111/1365-2745.12078.

[ece374128-bib-0023] Fulcher, M. R. , and R. C. Little . 2024. “Development and Application of the Fungal Plant Pathogen Colletotrichum Shisoi to Control Invasive *Perilla frutescens* .” Biological Control 194: 105543. 10.1016/j.biocontrol.2024.105543.

[ece374128-bib-0024] Fulcher, M. R. , M. A. Tancos , R. C. Mueller , and M. Tannières . 2024. “Importance of Pathobiomes to the Success of Microbial Weed Biocontrol Agents.” Biological Control 192: 105498. 10.1016/j.biocontrol.2024.105498.

[ece374128-bib-0025] Fulcher, M. R. , A. Tritz , V. Beauchamp , and C. A. Wu . 2026. “Wavyleaf Basketgrass ( *Oplismenus undulatifolius* ) Invasion Is Associated With Changes in Soil Microbial Communities.” mSphere 11: e00895‐25. 10.1128/msphere.00895-25. 42053319 PMC13203986

[ece374128-bib-0026] Gohl, D. M. , P. Vangay , J. Garbe , et al. 2016. “Systematic Improvement of Amplicon Marker Gene Methods for Increased Accuracy in Microbiome Studies.” Nature Biotechnology 34: 942–949. 10.1038/nbt.3601. 27454739

[ece374128-bib-0027] Gundale, M. J. , J. P. Almeida , H. Wallander , et al. 2016. “Differences in Endophyte Communities of Introduced Trees Depend on the Phylogenetic Relatedness of the Receiving Forest.” Journal of Ecology 104: 1219–1232. 10.1111/1365-2745.12595.

[ece374128-bib-0028] Hardoim, P. R. , L. S. Van Overbeek , G. Berg , et al. 2015. “The Hidden World Within Plants: Ecological and Evolutionary Considerations for Defining Functioning of Microbial Endophytes.” Microbiology and Molecular Biology Reviews 79: 293–320. 10.1128/MMBR.00050-14. 26136581 PMC4488371

[ece374128-bib-0029] Hoddle, M. S. 2024. “A New Paradigm: Proactive Biological Control of Invasive Insect Pests.” BioControl 69: 321–334. 10.1007/s10526-023-10206-5.

[ece374128-bib-0030] Hornung, B. V. H. , R. D. Zwittink , and E. J. Kuijper . 2019. “Issues and Current Standards of Controls in Microbiome Research.” FEMS Microbiology Ecology 95: fiz045. 10.1093/femsec/fiz045. 30997495 PMC6469980

[ece374128-bib-0031] Hsieh, T. C. , K. H. Ma , and A. Chao . 2016. “iNEXT: An R Package for Rarefaction and Extrapolation of Species Diversity (Hill Numbers).” Methods in Ecology and Evolution 7: 1451–1456. 10.1111/2041-210X.12613.

[ece374128-bib-0032] Keane, R. M. , and M. J. Crawley . 2002. “Exotic Plant Invasions and the Enemy Release Hypothesis.” Trends in Ecology & Evolution 17: 164–170. 10.1016/S0169-5347(02)02499-0.

[ece374128-bib-0033] Kendig, A. E. , V. J. Svahnström , A. Adhikari , P. F. Harmon , and S. L. Flory . 2021. “Emerging Fungal Pathogen of an Invasive Grass: Implications for Competition With Native Plant Species.” PLoS One 16: 1–17. 10.1371/journal.pone.0237894. PMC7920361 33647021

[ece374128-bib-0034] Kepner, C. , and V. B. Beauchamp . 2020. “Interspecific Competitive Potential of Wavyleaf Basketgrass ( *Oplismenus undulatifolius* ), a Recent Introduction to the Mid‐Atlantic United States.” Invasive Plant Science and Management 13: 23–29. 10.1017/inp.2020.3.

[ece374128-bib-0035] Kowalski, K. P. , C. Bacon , W. Bickford , et al. 2015. “Advancing the Science of Microbial Symbiosis to Support Invasive Species Management: A Case Study on Phragmites in the Great Lakes.” Frontiers in Microbiology 6: 1–14. 10.3389/fmicb.2015.00095. 25745417 PMC4333861

[ece374128-bib-0036] Kuebbing, S. E. , M. A. Nuñez , and D. Simberloff . 2013. “Current Mismatch Between Research and Conservation Efforts: The Need to Study Co‐Occurring Invasive Plant Species.” Biological Conservation 160: 121–129. 10.1016/j.biocon.2013.01.009.

[ece374128-bib-0037] LaForgia, M. L. , H. Kang , and C. L. Ettinger . 2022. “Invasive Grass Dominance Over Native Forbs Is Linked to Shifts in the Bacterial Rhizosphere Microbiome.” Microbial Ecology 84: 496–508. 10.1007/s00248-021-01853-1. 34505915 PMC9436828

[ece374128-bib-0038] Lane, B. R. , A. E. Kendig , C. M. Wojan , et al. 2023. “Fungicide‐Mediated Shifts in the Foliar Fungal Community of an Invasive Grass.” Phytobiomes Journal 7: 198–207. 10.1094/PBIOMES-03-22-0018-R.

[ece374128-bib-0039] Lane, B. R. , M. A. Kuhs , M. M. Zaret , et al. 2025. “Foliar Fungi‐Imposed Costs to Plant Productivity Moderate Shifts in Composition of the Rhizosphere Microbiome.” Frontiers in Plant Science 16: 1558191. 10.3389/fpls.2025.1558191. 40110355 PMC11921152

[ece374128-bib-0040] Lenth, R. V. , and J. Piaskowski . 2026. “emmeans: Estimated Marginal Means, aka Least‐Squares Means.” v 2.0.4. 10.32614/CRAN.package.emmeans.

[ece374128-bib-0041] Liang, Y. , D. Ning , Z. Lu , et al. 2020. “Century Long Fertilization Reduces Stochasticity Controlling Grassland Microbial Community Succession.” Soil Biology and Biochemistry 151: 108023. 10.1016/j.soilbio.2020.108023.

[ece374128-bib-0042] Lin, H. , and S. D. Peddada . 2020. “Analysis of Compositions of Microbiomes With Bias Correction.” Nature Communications 11: 3514. 10.1038/s41467-020-17041-7. PMC7360769 32665548

[ece374128-bib-0043] Lu‐Irving, P. , J. G. Harenčár , H. Sounart , et al. 2019. “Native and Invading Yellow Starthistle ( *Centaurea solstitialis* ) Microbiomes Differ in Composition and Diversity of Bacteria.” mSphere 4: e00088‐19. 10.1128/mSphere.00088-19. 30842267 PMC6403453

[ece374128-bib-0044] Martin, M. 2011. “Cutadapt Removes Adapter Sequences From High‐Throughput Sequencing Reads.” EMBnet.Journal 17: 10–12. 10.14806/ej.17.1.200.

[ece374128-bib-0045] McDonald, D. , Y. Jiang , M. Balaban , et al. 2024. “Greengenes2 Unifies Microbial Data in a Single Reference Tree.” Nature Biotechnology 42: 715–718. 10.1038/s41587-023-01845-1. PMC10818020 37500913

[ece374128-bib-0046] Mei, L. , M. Zhu , D.‐Z. Zhang , Y. Z. Wang , J. Guo , and H. B. Zhang . 2014. “Geographical and Temporal Changes of Foliar Fungal Endophytes Associated With the Invasive Plant *Ageratina adenophora* .” Microbial Ecology 67: 402–409. 10.1007/s00248-013-0319-8. 24276537

[ece374128-bib-0047] Mitchell, C. E. , A. A. Agrawal , J. D. Bever , et al. 2006. “Biotic Interactions and Plant Invasions.” Ecology Letters 9: 726–740. 10.1111/j.1461-0248.2006.00908.x. 16706916

[ece374128-bib-0048] Mitchell, C. E. , D. Blumenthal , V. Jarošík , E. E. Puckett , and P. Pyšek . 2010. “Controls on Pathogen Species Richness in Plants' Introduced and Native Ranges: Roles of Residence Time, Range Size and Host Traits.” Ecology Letters 13: 1525–1535. 10.1111/j.1461-0248.2010.01543.x. 20973907 PMC3003901

[ece374128-bib-0049] Moles, A. T. , R. L. Dalrymple , S. Raghu , S. P. Bonser , and J. Ollerton . 2022. “Advancing the Missed Mutualist Hypothesis, the Under‐Appreciated Twin of the Enemy Release Hypothesis.” Biology Letters 18: 20220220. 10.1098/rsbl.2022.0220. 36259169 PMC9579764

[ece374128-bib-0050] Morin, L. 2020. “Progress in Biological Control of Weeds With Plant Pathogens.” Annual Review of Phytopathology 58: 201–223. 10.1146/annurev-phyto-010820-012823. 32384863

[ece374128-bib-0051] Najberek, K. , A. Olszańska , B. Tokarska‐Guzik , K. Mazurska , Z. Dajdok , and W. Solarz . 2022. “Invasive Alien Species as Reservoirs for Pathogens.” Ecological Indicators 139: 108879. 10.1016/j.ecolind.2022.108879.

[ece374128-bib-0052] Nakamura, N. , H. Toju , and K. Kitajima . 2023. “Leaf, Root, and Soil Microbiomes of an Invasive Plant, *Ardisia crenata* , Differ Between Its Native and Exotic Ranges.” Frontiers in Microbiology 14: 1302167. 10.3389/fmicb.2023.1302167. 38075909 PMC10703299

[ece374128-bib-0053] Ning, D. , Y. Deng , J. M. Tiedje , and J. Zhou . 2019. “A General Framework for Quantitatively Assessing Ecological Stochasticity.” Proceedings of the National Academy of Sciences 116: 16892–16898. 10.1073/pnas.1904623116. PMC6708315 31391302

[ece374128-bib-0054] Oksanen, J. , F. G. Blanchet , M. Friendly , et al. 2026. “Vegan: Community Ecology Package.” v2.7. 10.32614/CRAN.package.vegan.

[ece374128-bib-0055] Pan, Y. , M. Liu , A. Sosa , B. Li , M. Shi , and X. Pan . 2023. “Hierarchical Metacommunity Structure of Fungal Endophytes.” New Phytologist 239: 1464–1474. 10.1111/nph.19065. 37292017

[ece374128-bib-0056] Pedregosa, F. , G. Varoquaux , A. Gramfort , et al. 2011. “Scikit‐Learn: Machine Learning in Python.” Journal of Machine Learning Research 12: 2825–2830.

[ece374128-bib-0057] Pham, D. H. , and C. A. Wu . 2023. “Seed Longevity and Germination of the Emerging Invasive Species Wavyleaf Basketgrass ( *Oplismenus undulatifolius* ) Under Varied Light Regimes.” Invasive Plant Science and Management 16, no. 4: 225–232. 10.1017/inp.2023.27.

[ece374128-bib-0058] Pickett, S. T. A. 1989. “Space‐For‐Time Substitution as an Alternative to Long‐Term Studies.” In Long‐Term Studies in Ecology, edited by G. E. Likens , 110–135. Springer New York.

[ece374128-bib-0061] Posit Team . 2025. “RStudio: Integrated Development Environment for R.” v.2025.05.0. Posit Software, PBC. http://www.posit.co/.

[ece374128-bib-0059] R Core Team . 2025. “R: A Language and Environment for Statistical Computing.” v4.4.3. R Foundation for Statistical Computing. https://www.R‐project.org/.

[ece374128-bib-0060] Rognes, T. , T. Flouri , B. Nichols , C. Quince , and F. Mahé . 2016. “VSEARCH: A Versatile Open Source Tool for Metagenomics.” PeerJ 4: e2584. 10.7717/peerj.2584. 27781170 PMC5075697

[ece374128-bib-0062] Schloss, P. D. 2024. “Rarefaction Is Currently the Best Approach to Control for Uneven Sequencing Effort in Amplicon Sequence Analyses.” mSphere 9: e0035423. 10.1128/msphere.00354-23. 38251877 PMC10900887

[ece374128-bib-0071] Shahrtash, M. , and S. P. Brown . 2020. “Drivers of Foliar Fungal Endophytic Communities of Kudzu (*Pueraria montana* var. *lobata*) in the Southeast United States.” Diversity 12, no. 5: 185. 10.3390/d12050185.

[ece374128-bib-0063] Shipunov, A. , G. Newcombe , A. K. H. Raghavendra , and C. L. Anderson . 2008. “Hidden Diversity of Endophytic Fungi in an Invasive Plant.” American Journal of Botany 95: 1096–1108. 10.3732/ajb.0800024. 21632429

[ece374128-bib-0064] Smith, M. C. , K. Canavan , C. R. Minteer , and D. Lieurance . 2025. “Preemptive and Proactive Application of Biological Control for Weeds: An Argument for Swifter Action to Aid Conservation Efforts.” Biological Control 202: 105725. 10.1016/j.biocontrol.2025.105725.

[ece374128-bib-0065] Stricker, K. B. , P. F. Harmon , E. M. Goss , K. Clay , and S. Luke Flory . 2016. “Emergence and Accumulation of Novel Pathogens Suppress an Invasive Species.” Ecology Letters 19: 469–477. 10.1111/ele.12583. 26931647

[ece374128-bib-0066] Taylor, J. E. , K. D. Hyde , and E. B. G. Jones . 1999. “Endophytic Fungi Associated With the Temperate Palm, *Trachycarpus fortunei* , Within and Outside Its Natural Geographic Range.” New Phytologist 142: 335–346. 10.1046/j.1469-8137.1999.00391.x.

[ece374128-bib-0067] Trognitz, F. , E. Hackl , S. Widhalm , and A. Sessitsch . 2016. “The Role of Plant—Microbiome Interactions in Weed Establishment and Control.” FEMS Microbiology Ecology 92: fiw138. 10.1093/femsec/fiw138. 27387910

[ece374128-bib-0068] Visscher, M. , and I. Dickie . 2021. “Native and Exotic Grasses Share Generalist Foliar Fungi in a Canterbury High Country Grassland.” New Zealand Journal of Ecology 45: 3451. 10.20417/nzjecol.45.23.

[ece374128-bib-0069] White, T. J. , T. Bruns , S. Lee , and J. Taylor . 1990. “Amplification and Direct Sequencing of Fungal Ribosomal RNA Genes for Phylogenetics.” In PCR Protocols, 315–322. Elsevier.

