# Supplementary material for: Differentiation of the Foliar Microbiomes From Co‐Occurring Grass Species Reflects Their Recency of Invasion
Source: Ecol Evol. 2026 Aug 7;16(8):e74128. doi: 10.1002/ece3.74128 (PMC13451155; doi:10.1002/ece3.74128)
Supplement: Supplementary file 1 [file ECE3-16-e74128-s001.docx]

**Appendix**

**Table S1.** Summary of analysis of variance to test for the significance of host effect in mixed linear models of microbial alpha diversity

| **Marker** | **Depth^a^** | **q = 0^b^** | **q = 1** | **q = 2** |
| --- | --- | --- | --- | --- |
| ITS | 10 | χ² = 14.3, p < 0.001^c^ | χ² = 12.5, p < 0.001 | χ² = 10.9, p < 0.001 |
| ITS | 100 | χ² = 38.7, p < 0.001 | χ² = 18.2, p < 0.001 | χ² = 11.3, p < 0.001 |
| ITS | 400 | χ² = 43.6, p < 0.001 | χ² = 19.8, p < 0.001 | χ² = 11.8, p < 0.001 |
| ITS | 700 | χ² = 43.1, p < 0.001 | χ² = 16.4, p < 0.001 | χ² = 8.89, p = 0.003 |
| ITS | 1000 | χ² = 28.4, p < 0.001 | χ² = 8.63, p = 0.003 | χ² = 4.28, p = 0.039 |
| ITS | 1300 | χ² = 26.5, p < 0.001 | χ² = 9.22, p = 0.002 | χ² = 4.95, p = 0.026 |
| ITS | 1600 | χ² = 22.6, p < 0.001 | χ² = 10.0, p = 0.002 | χ² = 6.06, p = 0.014 |
| ITS | 1900 | χ² = 22.4, p < 0.001 | χ² = 11.8, p < 0.001 | χ² = 7.33, p = 0.007 |
| 16S | 10 | χ² = 28.8, p < 0.001 | χ² = 30.3, p < 0.001 | χ² = 29.5, p < 0.001 |
| 16S | 100 | χ² = 4.25, p = 0.039 | χ² = 18.0, p < 0.001 | χ² = 22.5, p < 0.001 |
| 16S | 400 | χ² = 2.17, p = 0.140 | χ² = 13.8, p < 0.001 | χ² = 20.2, p < 0.001 |
| 16S | 700 | χ² = 1.23, p = 0.268 | χ² = 9.98, p = 0.002 | χ² = 13.8, p < 0.001 |
| 16S | 1000 | χ² = 0.10, p = 0.754 | χ² = 6.72, p = 0.010 | χ² = 7.95, p = 0.005 |
| 16S | 1300 | χ² = 0.73, p = 0.392 | χ² = 7.44, p = 0.006 | χ² = 8.71, p = 0.003 |
| 16S | 1600 | χ² = 1.15, p = 0.284 | χ² = 6.01, p = 0.014 | χ² = 6.96, p = 0.008 |
| 16S | 1900 | χ² = 2.46, p = 0.117 | χ² = 7.11, p = 0.008 | χ² = 8.23, p = 0.004 |

^a^Read depth for rarefied diversity estimates

^b^Diversity estimated as effective number of OTUs, or Hill numbers order q = 0 to 2, corresponding to richness, exponential Shannon Diversity, and inverse Simpson Diversity

^c^Wald chi-square test for significance of host species identity as a predictor

**Table S2.** Summary of linear mixed model parameter estimates for alpha diversity across rarefaction depths.

| **Marker** | **Depth^a^** | **q=0^b^** | | | | |
| --- | --- | --- | --- | --- | --- | --- |
|  |  | **Random Plot Effect** | | **Fixed Host Effects^d^** | | |
|  |  | **Variance^c^** | **Residual** | **Estimate** | **SE** | **t** |
| ITS | 10 | 0 | 0.098 | 0.211 | 0.056 | 3.78 |
| ITS | 100 | 0.006 | 0.13 | 0.4 | 0.064 | 6.22 |
| ITS | 400 | 0.013 | 0.134 | 0.44 | 0.067 | 6.6 |
| ITS | 700 | 0.017 | 0.128 | 0.455 | 0.069 | 6.56 |
| ITS | 1000 | 0.021 | 0.115 | 0.38 | 0.071 | 5.32 |
| ITS | 1300 | 0.021 | 0.116 | 0.371 | 0.072 | 5.14 |
| ITS | 1600 | 0.023 | 0.112 | 0.347 | 0.073 | 4.75 |
| ITS | 1900 | 0.024 | 0.111 | 0.352 | 0.074 | 4.73 |
| 16S | 10 | 0.006 | 0.15 | 0.371 | 0.069 | 5.36 |
| 16S | 100 | 0.016 | 0.292 | 0.199 | 0.097 | 2.06 |
| 16S | 400 | 0.008 | 0.27 | -0.141 | 0.095 | -1.47 |
| 16S | 700 | 0.009 | 0.286 | -0.13 | 0.117 | -1.11 |
| 16S | 1000 | 0 | 0.294 | 0.048 | 0.152 | 0.31 |
| 16S | 1300 | 0 | 0.288 | 0.151 | 0.176 | 0.86 |
| 16S | 1600 | 0 | 0.287 | 0.216 | 0.202 | 1.07 |
| 16S | 1900 | 0 | 0.265 | 0.323 | 0.206 | 1.57 |
|  |  | **q=1** | | | | |
| ITS | 10 | 0 | 0.133 | 0.23 | 0.065 | 3.53 |
| ITS | 100 | 0 | 0.235 | 0.369 | 0.086 | 4.27 |
| ITS | 400 | 0 | 0.244 | 0.398 | 0.09 | 4.45 |
| ITS | 700 | 0.002 | 0.233 | 0.378 | 0.093 | 4.05 |
| ITS | 1000 | 0.003 | 0.21 | 0.281 | 0.096 | 2.94 |
| ITS | 1300 | 0.003 | 0.211 | 0.294 | 0.097 | 3.04 |
| ITS | 1600 | 0.003 | 0.213 | 0.314 | 0.099 | 3.17 |
| ITS | 1900 | 0.004 | 0.21 | 0.345 | 0.101 | 3.43 |
| 16S | 10 | 0.011 | 0.207 | 0.447 | 0.081 | 5.5 |
| 16S | 100 | 0.032 | 0.572 | 0.573 | 0.135 | 4.24 |
| 16S | 400 | 0.029 | 0.706 | 0.573 | 0.154 | 3.71 |
| 16S | 700 | 0.047 | 0.896 | 0.657 | 0.208 | 3.16 |
| 16S | 1000 | 0.058 | 1.047 | 0.757 | 0.292 | 2.59 |
| 16S | 1300 | 0.082 | 1.083 | 0.945 | 0.346 | 2.73 |
| 16S | 1600 | 0.069 | 1.16 | 1.006 | 0.41 | 2.45 |
| 16S | 1900 | 0.083 | 1.15 | 1.155 | 0.433 | 2.67 |
|  |  | **q=2** | | | | |
| ITS | 10 | 0 | 0.136 | 0.217 | 0.066 | 3.3 |
| ITS | 100 | 0 | 0.242 | 0.295 | 0.088 | 3.36 |
| ITS | 400 | 0 | 0.245 | 0.307 | 0.09 | 3.43 |
| ITS | 700 | 0 | 0.23 | 0.277 | 0.093 | 2.98 |
| ITS | 1000 | 0 | 0.214 | 0.199 | 0.096 | 2.07 |
| ITS | 1300 | 0 | 0.213 | 0.215 | 0.097 | 2.22 |
| ITS | 1600 | 0 | 0.211 | 0.242 | 0.098 | 2.46 |
| ITS | 1900 | 0 | 0.208 | 0.269 | 0.1 | 2.71 |
| 16S | 10 | 0.013 | 0.227 | 0.462 | 0.085 | 5.43 |
| 16S | 100 | 0.035 | 0.576 | 0.643 | 0.136 | 4.74 |
| 16S | 400 | 0.032 | 0.684 | 0.683 | 0.152 | 4.5 |
| 16S | 700 | 0.044 | 0.865 | 0.759 | 0.204 | 3.72 |
| 16S | 1000 | 0.057 | 0.997 | 0.804 | 0.285 | 2.82 |
| 16S | 1300 | 0.076 | 1.013 | 0.988 | 0.335 | 2.95 |
| 16S | 1600 | 0.062 | 1.072 | 1.04 | 0.394 | 2.64 |
| 16S | 1900 | 0.072 | 1.061 | 1.193 | 0.416 | 2.87 |

^a^Read depth for rarefied diversity estimates

^b^Diversity estimated as effective number of OTUs, or Hill numbers order q = 0 to 2, corresponding to richness, exponential Shannon Diversity, and inverse Simpson Diversity

^c^Variance estimates of 0 indicate singular model fits where sample location did not account for detectable variation in data. These model outputs were retained for consistency across depths and markers.

^d^Estimated effect of stiltgrass host identity relative to basketgrass

**Fig. S1. Rarefaction curves for foliar microbial diversity in *Oplismenus* and *Microstegium***

Diversity of **(a)** fungal (ITS) and **(b)** bacterial (16S) sequences from endophytic communities in basketgrass and stiltgrass leaves. The effective number of OTUs, or Hill numbers of order q=0 (richness), q=1 (exponential Shannon Diversity), and q=2 (inverse Simpson Diversity), were estimated across a range of rarefaction depths based on bootstrap resampling (n=100 per depth). Each line represents the community from a single leaf. Shading denotes host.
